# Sequence-specific thermodynamic properties of nucleic acids influence both transcriptional pausing and backtracking in yeast

**DOI:** 10.1371/journal.pone.0174066

**Published:** 2017-03-16

**Authors:** Martin Lukačišin, Matthieu Landon, Rishi Jajoo

**Affiliations:** 1 Department of Systems Biology, Harvard Medical School, Boston, MA, United States of America; 2 IST Austria, Klosterneuburg, Austria; 3 Department of Genetics, Harvard Medical School, Boston, MA, United States of America; 4 Ecole des Mines de Paris, Mines Paristech, Paris, France; Southern Illinois University School of Medicine, UNITED STATES

## Abstract

RNA Polymerase II pauses and backtracks during transcription, with many consequences for gene expression and cellular physiology. Here, we show that the energy required to melt double-stranded nucleic acids in the transcription bubble predicts pausing in *Saccharomyces cerevisiae* far more accurately than nucleosome roadblocks do. In addition, the same energy difference also determines when the RNA polymerase backtracks instead of continuing to move forward. This data-driven model corroborates—in a genome wide and quantitative manner—previous evidence that sequence-dependent thermodynamic features of nucleic acids influence both transcriptional pausing and backtracking.

## Introduction

RNA polymerase II (RNAP) transcribes intermittently, pausing and backtracking along DNA before continuing to transcribe [1,2]. Transcriptional pausing is a critical feature of gene regulation [3] and is involved in RNA splicing [4], transcription fidelity [5] and transcription termination [6] and has been implicated in genome instability [7]. Recently however, a new technique for determining RNAP occupancy across the genome (NET-seq: Native Elongating Transcript sequencing) has been developed. In this technique, RNAP molecules from flash-frozen cells are isolated, and the 3’ end of the nascent RNA associated with RNAP is sequenced. Mapping of this sequence to the genome thus reveals information about the position of RNAP [8]. NET-seq has revealed that RNAP pauses and backtracks reproducibly at over 10^5^ specific locations in the *Saccharomyces cerevisiae* genome [8]. The causes and functions of these pausing and backtracking events are not yet fully understood.

Several factors have been demonstrated to have an influence on RNAP pausing and backtracking. Nucleosomes have been shown to elicit RNAP pausing in single-molecule studies [9] and also show correlation with high-throughput pausing data [8]. Similarly, transcription factors bound to DNA act as roadblocks to RNA polymerase [10]. Interaction between nucleic acids and the residues from RNAP influence both RNAP pausing [5,11] and backtracking [12], as does the structure of the nascent RNA [13,14]. Last but not least, the basepairing strength of the nucleic acids inside the transcription bubble has been shown to be a determinant of transcriptional pausing experimentally [15,16] and this dependence has been further explored using thermodynamic [17] and kinetic models [18].

It is difficult, however, to quantitatively disentangle the contribution of these factors to the wide-spread RNAP pausing. In the present study, we made use of publicly available genome-wide datasets of RNAP pausing and backtracking [8] and nucleosome positions [19] to compare the explanatory power of basepairing thermodynamics of nucleic acids and known nucleosome positions on the reported pervasive RNAP pausing and backtracking [8]. To this end, we also built a simple, data-driven quantitative model of how basepairing of nucleic acids affects RNAP pausing during forward movement, pausing during backtracking and the incidence of backtracks.

## Results

### Currently mapped nucleosomes explain much less pausing than nucleotide sequence

In the elongation phase of transcription, RNAP transcribes intermittently and RNAP pausing is often followed by backtracking. During backtracking, RNAP moves upstream of the initial pause site and the 3' end of the nascent RNA comes out of the RNAP active site into the RNAP backtracking channel [2,8,12]. The elongation factor TFIIS, encoded by the gene *DST1* in *S*. *cerevisiae*, then stimulates RNAP to cleave the backtracked 3' stretch of the nascent RNA, allowing transcription to restart from an upstream point. NET-seq allows genome-wide, single-base detection of both the initial sites of polymerase pausing and the subsequent sites of nascent transcript cleavage. When performed on WT cells, NET-seq reports the backtracked and post-cleaved state, upstream of the initial pause; in *dst1Δ* strains, due to the lack of TFIIS, the 3' end of nascent transcripts is left intact, and NET-seq reveals the site where RNAP paused while polymerizing RNA [8], irrespective of whether RNAP subsequently backtracked or not. In this work, we use the term “initial pause” to refer to the position of the 3’ RNA nucleotide sequenced in the *dst1Δ* strain and “backtracked pause” to refer to the 3’ RNA nucleotide sequenced in the WT strain.

**Fig 1:**
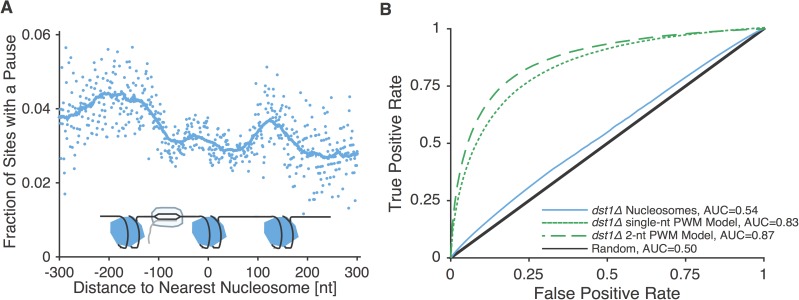
Mapped nucleosome positions explain only a small portion of RNAP pausing. **A.** Fraction of sites with a pause as detected via NET-seq in *dst1Δ* yeast strains as a function of distance from the nearest annotated nucleosome. The trendline represent a 61 base-pair running average. **B.** Receiver operating characteristic curves and AUC values for predictions based on nucleosome positions, a single-base position weight matrix and a nearest-neighbor position weight matrix.

Because nucleosomes wound on DNA have previously been investigated as “roadblocks” that cause RNAP pausing [8,20–22], we tested whether measured nucleosome positions could predict the sites where RNAP paused. Specifically, nucleosomes are thought to impede polymerases during downstream movement [8], so we used a single base-pair map of nucleosome positions [19] and aligned it against RNAP pause locations from *dst1Δ* NET-seq data [8]. As previously observed [8], RNAP pausing increases near nucleosomes (Fig 1A).

To better quantify how well nucleosome positions explained RNAP pausing, we used the Area Under the Receiver Operating Characteristic curve (AUC, which takes the values 1.0 for perfect prediction and 0.5 for uninformed guessing): we fitted the pause frequency near nucleosomes to predict RNAP pausing and found an AUC value of 0.54 (Fig 1B), indicating that the mapped nucleosomes only explain a small fraction of pausing in *S*. *cerevisiae*. Since the prediction proceeds from fitting pausing to nucleosome positions, this limited predictive power is likely the upper bound for known nucleosome location on RNAP pausing. It is worth noting that this result is consistent both with nucleosomes not having a strong influence on RNAP pausing as well as with nucleosomes being a strong inducer of pausing but most pauses not being caused by the currently mapped nucleosomes. Most importantly, the low frequency of nucleosomes relative to the number of pauses accounts for their low predictive power. Because the same RNAP pause sites are seen in different cells and in different biological replicates [8], it is unlikely that nucleosomes without strong positional preference could cause a major fraction of RNAP pausing.

Next, we examined how initial pausing (i.e. *dst1Δ* NET-seq data) was influenced by DNA sequence at the site of the pause. To do this we aggregated and aligned the sequences surrounding each initial pause site to obtain a position weight matrix (PWM, see Materials and Methods), a commonly used representation of DNA sequence motifs [23]. We then scored how efficiently DNA sequence at all transcribed locations correlated with the known locations of RNAP pausing. This analysis is similar to the one performed on the NET-seq data by Churchman and Weissman [8].

We found that nucleotide sequence (extracted from the PWM) could predict pausing with an AUC of 0.83, far more than the nucleosome positions could (Fig 1B). The explanatory power of the sequence increased further when accounting for possible nearest neighbor nucleotide interactions, to an AUC of 0.87 (Fig 1B; see Materials and Methods). Since, however, using the PWM on its own as a predictor offers little biological insight into the mechanism of pausing, we sought to rationalize the sequence dependence of RNAP pausing.

### Dependence of pausing on nucleotide sequence can be rationalized in terms of basepairing energy

**Fig 2:**
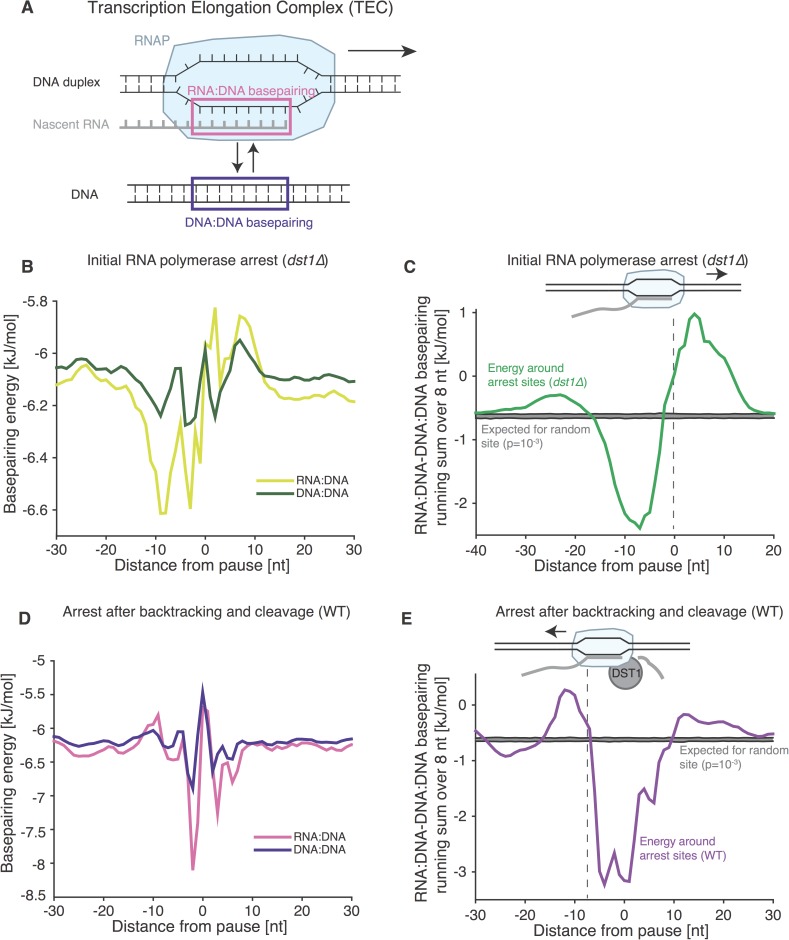
RNA:DNA and DNA:DNA basepairing energy change near pause sites. **A.** The stability of transcriptional elongation complex is influenced by sequence-dependent RNA:DNA and DNA:DNA basepairing energies. **B.** Average basepairing energy for RNA:DNA and DNA:DNA duplexes, around initial transcriptional arrest sites in a *dst1Δ* strain. Each pause site is aligned and the basepairing energy around it is averaged for each position separately over all pause sites considered. **C.** The difference between RNA:DNA and DNA:DNA basepairing around *dst1Δ* pause sites plotted as a running sum over the presumed length of RNA:DNA hybrid (8 bp). Each pause site is aligned and the basepairing energy for a stretch of 60 bases around it is summed with a centered sliding window of 8 bases and averaged over all pause sites considered. The expectation for random sites in the transcriptome is plotted at the p = 10^−3^ level (gray area). **D.-E.** Same as (B) and (C), respectively, but for sites of transcriptional arrest as determined from NET-seq in WT strains where RNAP is likely to be pausing while moving upstream. The dashed line marks the front end of the RNA:DNA hybrid in the direction of RNAP movement (arrow).

We reasoned that pausing could depend on sequence if RNAP thermodynamically favored its current position more than a downstream position, based on the differences in basepairing strength for both the DNA:DNA duplex of the genome and the RNA:DNA duplex formed inside RNAP, as suggested in previous studies [15–18,24]. We noted that even though RNA:DNA basepairing is slightly more energetically favorable *on average* than DNA:DNA basepairing, there are many sequences for which the opposite is true, allowing specific sequences to prefer RNA:DNA or DNA:DNA basepairing. We then calculated the average basepairing energy for both DNA:DNA and RNA:DNA around all pause sites one basepair at a time. To do this, we used a sequence-dependent nearest neighbor model as is often used to determine PCR primer melting temperatures [25,26] (see Materials and Methods and S1 and S2 Tables). We then averaged the melting energy for the positions around all pause sites to determine if any systematic pattern emerged (Fig 2C and 2E).

In the *dst1Δ* NET-seq dataset—which captures the position where RNAP pauses when moving downstream, (i.e. in the direction of transcription)—we found that the sequences directly upstream of pause sites have more stable RNA:DNA basepairing than DNA:DNA basepairing (Fig 2A and 2B). These are the nucleotides which would form the RNA:DNA hybrid inside RNAP. Further, in the sequence directly downstream of pause sites, RNA:DNA basepairing is weaker than DNA:DNA basepairing. These are the DNA:DNA base pairs that would need to be melted for the polymerase to continue moving downstream. Although these bases have not yet been transcribed, they seem to influence pausing; this observation will be discussed further below. Therefore, we suggest that the polymerase is found at specific locations in NET-seq data because it thermodynamically favors these positions over positions downstream.

For the WT dataset, where the polymerase is assumed to be backtracking, RNA:DNA basepairing is weaker than DNA:DNA basepairing in the sequence directly *upstream* (i.e. in the direction of backtracking) of RNAP, suggesting that RNAP pauses during backtracking at positions it thermodynamically favors over continuing its backtracking. These are the sites where subsequent cleavage mediated by TFIIS is most likely to occur, and thus these sites are revealed in the WT NET-seq dataset.

**Fig 3:**
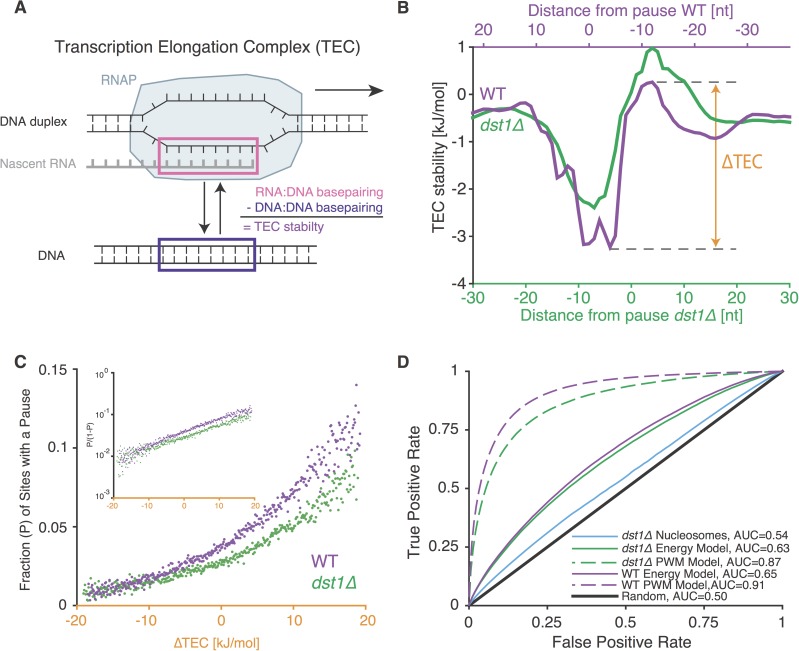
Change in the difference between RNA:DNA and DNA:DNA basepairing strength is a good predictor of RNAP pausing. **A.** The transcription elongation complex (TEC) contains an RNA:DNA hybrid that takes the place of a DNA:DNA duplex as the polymerase transcribes; we define the TEC stability as the difference between the energy required to melt these two structures. **B.** Assuming an 8 bp long RNA:DNA hybrid, the average TEC stability is plotted around *dst1Δ* and WT pause site as in Fig 2C and 2E. Note that WT data is plotted on an inverted and shifted secondary axis. This shows the similarity between the TEC stability profiles if we assume that RNAP is moving upstream when captured by WT NET-seq and moving downstream in *dst1Δ* NET-seq. **C.** The fraction of sites with a pause for the highly expressed genes (see Materials and Methods) as a function of TEC stability energy difference at those sites (ΔTEC, as defined in B). Inset: Same data with a logarithmic axis to show that the odds of pausing increase exponentially with ΔTEC. **D.** Receiver operating characteristic curves and AUC values for transcription pause sites using different models. The curves for position weight matrix (PWM) models are those for nearest-neighbor PWMs to ensure fair comparison with the energy model which also considers nearest neighbor interactions.

If the difference in RNA:DNA and DNA:DNA basepairing strength is summed over the presumed length of RNA:DNA hybrid of 8 bp [5,12,15,27] (for simplicity, we assumed the length of RNA:DNA hybrid to be constant), a clear pattern emerges for both *dst1Δ* and WT datasets (Fig 2C and 2E). Based on the direction of RNA polymerase movement, the polymerase pauses at sites when the change in RNA:DNA basepairing energy compared to DNA:DNA basepairing energy is greatest. Importantly, the change in basepairing energies around pause sites for *dst1Δ* and WT datasets looks similar if the change in direction of RNAP movement is accounted for (Fig 3B). Specifically, in *dst1Δ* datasets, it is thermodynamically unfavorable for RNAP to move *downstream*, and in WT datasets it is thermodynamically unfavorable for RNAP to move *upstream*. To overlay the thermodynamic landscapes between RNAP downstream movement (*dst1Δ*) and upstream movement (WT), one has to switch the direction of the energy profiles and shift them by ~8 nt (Fig 3B). The distance between the 5’-end and the 3’-end of the RNA:DNA hybrid is ~8 bp, so this shift can be interpreted as an expected consequence of RNAP moving upstream, when the 3’-end rather than the 5’-end of the RNA:DNA hybrid is being unwound. Therefore RNAP likely pauses at specific sites due to the destabilisation of the TEC through weakened basepairing inside the TEC. Although possible, it is unlikely that transcription factors bound to DNA could also result in such a pattern of sequence-dependent pausing, since much of the sequence dependence comes from nucleotides that are buried within the RNAP.

### A simple thermodynamic basepairing energy model predicts pausing better than nucleosome positions do

To devise a simple proxy for the transcription elongation complex (TEC) stability that could predict pausing, we compute the difference between the stability of RNA:DNA hybrid and the stability of the same sequence if in a DNA:DNA duplex (Fig 3A). We used the difference between the TEC stability several bases upstream and downstream of each position (ΔTEC in Fig 3D, see Materials and Methods) to predict the probability of RNAP pausing at each position in the genome without fitting any parameters. Our simple metric omits the complex energy contribution caused by the unpaired DNA bases upstream and downstream of RNA:DNA hybrid interacting with the amino acids from RNAP. Nevertheless, the inferred TEC energy difference correlated extremely well with the likelihood of pauses in both the *dst1Δ* and WT NET-seq data (Fig 3C). The AUC for this model based only on the calculated basepairing energy (0.63) was higher than for the nucleosome model for *dst1Δ* (0.54). This difference allows greater than 20% more true positives at a given level of false negatives than the nucleosome model (Fig 3D). The model also captures a sizeable portion of all possible sequence related pausing as determined by the nearest neighbor PWM (0.87) (Fig 3D). The predictive capability of the PWM model represents a maximum for any sequence-related measure since it is built using direct sequence information from the pauses. Thus, there are likely other sequence-related effects included in the PWM which also influence RNAP dynamics and are absent in our energy model.

### The incidence of backtracks depends on nucleotide basepairing

Having determined a feature of DNA sequences that can predict RNAP pausing during downstream movement (*dst1Δ*) as well as during upstream movement (WT), we next asked whether this sequence feature could also predict the propensity of RNAP to enter into backtracking after an initial pause. This is of biological interest since RNAP backtracking increases transcriptional fidelity [1], but long backtracking excursions also promote genome instability [28].

**Fig 4:**
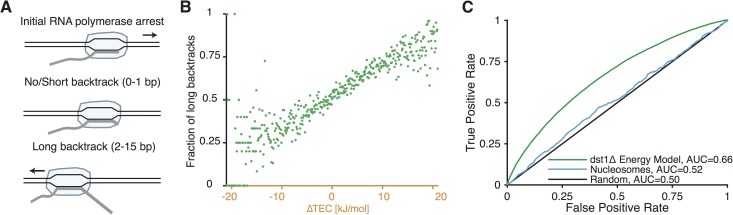
Energy barrier from basepairing positively correlates with long backtracks. **A.** After an initial pause, RNAP often backtracks. Pause sites from *dst1Δ* data were considered as leading to “no/short backtracking” if there was a corresponding pause site in the WT NET-seq dataset 0 or 1 bases upstream and as leading to “long backtracking” if the closest upstream WT pause site was 2 to 15 bases away. If there was no pause in the WT dataset in the region 0–15 bases upstream from the *dst1Δ* pause site, the *dst1Δ* pause was not included in the analysis. **B.** The fraction of *dst1Δ* pauses that lead to long backtracks (2 to 15bp) is plotted against the energy barrier as defined for *dst1Δ* in Fig 3C. **C.** Receiver operating characteristic curves and AUC values of predicting long backtracking in WT data from *dst1Δ* pause sites is shown for the energy model and for nucleosomes.

Since the *dst1Δ* NET-seq dataset shows the positions of initial pauses, and the WT NET-seq dataset shows the sites where the cleavage of 3’ end of backtracked RNA by TFIIS occurred [8], comparing the pause sites in these two datasets offers the opportunity to classify the pauses in *dst1Δ* the dataset as leading to backtracking or not. Pauses in *dst1Δ* the dataset for which there is a pause in the WT dataset that is 0–1 nt upstream were considered non-backtracking (63.9% of total *dst1Δ* pauses). Pauses in the *dst1Δ* the dataset for which there is a corresponding pause in the WT dataset between 2 and 15 nucleotides upstream were considered backtracking (35.7% of total *dst1Δ* pauses, Fig 4A). We found that pauses with higher ΔTEC were more likely to exhibit backtracking (Fig 4B). This correlation between higher ΔTEC and backtracking was far more predictive than nucleosome positions were (Fig 4C). Therefore, sequences with higher TEC stability differences not only cause more RNAP pauses, but also cause backtracking more often after the initial pause.

### Using basepairing energy model to estimate thermodynamic discrimination against transcriptional errors

RNA polymerase II has an estimated error rate of less than 10^−5^, much less than what would be expected by simple Watson-Crick basepairing [29,30]. RNAP achieves its additional fidelity using proofreading mechanisms including RNAP pausing and backtracking—incorporation of an incorrect RNA base triggers backtracking, followed by 3’ end cleavage and resynthesis of RNA. Error recognition followed by backtracking is reported to be based both on kinetic discrimination [5,29]—an incorrect base decreases the rate of addition of the next nucleotide—as well as on thermodynamic discrimination [1]—a mis-incorporated base has a low base-pairing energy, destabilizing the transcription elongation complex and biasing RNAP toward pausing and backtracking. We reasoned that this RNAP backtracking during the proofreading of a transcriptional error might also depend on the same basepairing thermodynamics as in our model.

We thus compared the ΔTEC energy that elicits a long backtrack (Fig 4), to the magnitude of thermodynamic destabilization of RNA:DNA hybrid by incorporation of an incorrect RNA base (S1 Fig). Predicted solely due to thermodynamic destabilization, RNAP is about 3-times as likely to pause and about 1.6-fold more likely to backtrack and perform TFIIS mediated 3’-end cleavage following the pause when there is an incorrect RNA base incorporated compared to the situation when there is no error. Consequently, RNAP would be about 5-times as likely to cleave a 3’ stretch of nascent RNA if it contains an incorrect base compared to a situation with no error, resulting in increased fidelity.

Such a rough comparison suggests that RNAP can indeed discriminate against transcriptional errors thermodynamically. However, the ~5-fold level of discrimination suggests that if TFIIS-dependent proofreading is to decrease RNAP error rate by e.g. 10%, i.e. 1 in 10 errors gets corrected, RNAP would have to pause and backtrack on average once every ~50 nt even in the absence of an error. Consequently, the reproducible, ubiquitous pausing of RNAP throughout the genome even in the absence of errors might be a trade-off for the contribution of TFIIS-dependent proofreading to the high fidelity of RNAP.

### Thermodynamic basepairing energy model is not confined to *S*. *cerevisiae*

As all DNA-dependent RNA polymerases have RNA:DNA duplexes, our energy model should also predict polymerase pausing in other contexts. Indeed, our TEC stability model also predicted transcriptional pausing in *E*. *coli* from bacterial NET-seq [31] with an AUC of 0.68 (S2 Fig). This indicates that in addition to effects unique to each species, changes in TEC stability due to underlying DNA sequence are a universal cause of RNA polymerase pausing during transcription.

## Discussion

In the present study we find that RNAP pauses at specific and ubiquitous DNA sequences for thermodynamic reasons. RNAP essentially operates as a Brownian ratchet [32]. As it transcribes and moves downstream, DNA:DNA base pairs ahead of the polymerase are broken and replaced with RNA:DNA base pairs inside of the transcription elongation complex (TEC). Upon further downstream movement, the RNA:DNA base pairs are broken as the nascent RNA exits from RNAP and the DNA:DNA duplex reforms. Since both RNA:DNA and DNA:DNA basepairing energies are sequence-specific, the propensity of RNA polymerase for forward movement is likely to be influenced by changes in thermodynamic stability due to the nucleotide sequence. By analyzing the pause sites uncovered in NET-seq datasets, we found that RNAP pauses where its current position in the *S*. *cerevisiae* genome is thermodynamically favored over the position further in its direction of movement. We find this to be the case both for RNAP pausing while the polymerase transcribes and moves downstream, as well as for RNAP pausing while the polymerase backtracks and thus moves in the upstream direction.

The similarity of basepairing energy profiles around pauses in WT and *dst1Δ* suggests that the underlying physical mechanism of RNAP pausing due to nucleotide sequence is the same irrespective of whether the polymerase moves forward (*dst1Δ* dataset) or backwards (WT dataset). It also provides further evidence that NET-seq of WT yeast strain captures the RNAP in the backtracked, post-cleavage state and thus that the restart of transcription after TFIIS cleavage is the rate limiting step during proofreading, as originally inferred only from the relative shifts between WT and *dst1Δ* NET-seq data [8].

It is intriguing to think that the RNA:DNA basepairing strength ahead of the actual position of RNA:DNA hybrid should influence RNA polymerase to stall as it does in our model, especially in the case of initial RNAP pausing while moving in 5’ to 3’ direction. One way for the RNAPII in a *dst1Δ* yeast strain to directly sample the RNA:DNA basepairing strength of this downstream stretch is to synthesize RNA over this region and then perform 3’→5’ cleavage using its intrinsic exonuclease activity even in the absence of TFIIS [33]. Alternatively, the basepairing strength might reflect an allosteric effect of the downstream DNA duplex being more difficult to unwind. The dependence of pausing on RNA:DNA basepairing strength is less surprising for pausing during backtracking, since RNAP in this case moves over a stretch of RNA already synthesized, and thus directly probes the differences in RNA:DNA basepairing over a given region.

The role of RNA:DNA hybrid strength in transcriptional processivity is well established [15,16,34]. However, here we find that in order to predict transcriptional pausing, the stability of the RNA:DNA hybrid *compared* to the stability of DNA:DNA duplex of the same sequence has to be considered. Such thermodynamic predictions are far more accurate than nucleosome positions at predicting where RNAP will pause. Moreover, the strength of the energy barrier causing the initial arrest of RNAP can predict how likely RNAP is to backtrack.

Other work has suggested specific kinetic landscapes to explain RNAP pausing and backtracking, often using single-molecule *in vitro* measurements of RNAP to inform or test the models [35]. In this work, we use *in vivo* data that is a snapshot over many different transcriptional events and therefore not directly comparable to kinetic models. Using NET-seq data and assuming that the number of 3’ ends of nascent RNA mapped to a location in the genome is proportional to the amount of time this nascent RNA exists during transcription (i.e. assuming ergodicity), we are instead able to more directly detect thermodynamic influences on RNAP dynamics [17] on the whole-genome level.

Other models have also suggested that the folding of nascent RNA as it exits the transcription bubble may influence the RNAP pausing and backtracking [36], however, our model did not examine this effect as the prediction of co-transcriptional folding of RNA has been challenging [37]. Recent work [38] could provide interesting directions for further study.

In addition, classifying pauses into short backtracks of 0–1 bp and long backtracks (2–15 bp), we find that long backtracks are more likely at positions where forward movement of RNAP is highly energetically unfavorable when considering the energetic costs of making and breaking the oligonucleotide duplexes required to continue forward movement. Since NET-seq is a snapshot technique and cannot directly measure the trajectories and lifetimes of single pauses and backtracks [39], further single-molecule experiments and biochemistry will be needed to continue validation of these ideas.

In sum, our results indicate that changes in the stability of the transcription elongation complex (TEC) based on sequence dependence of nucleotide basepairing is a more salient predictor of pausing than nucleosome positions. In addition, this measure is a good predictor of not only RNA polymerase pausing, but also of RNAP backtracking and we find a quantitative dependence between those features. However, the predictive power of our model is not total, meaning that other sequence-specific phenomena also play a role in transcriptional pausing and backtracking. Testing the predictions of our model and uncovering new sequence dependent determinants could be achieved by performing NET-seq on systematically recoded genes and genomes. Such iterations between computational predictions and experimental testing will paint a more complete picture of RNAP transcription, pausing and backtracking.

## Materials and methods

### Alignment

Sequencing data from Churchman et al. [8] was downloaded from http://www.ncbi.nlm.nih.gov/geo/ via GEO accession number GSE25107. Sequences were converted from.sra to fastq format with sratoolkit and were aligned to the SacCer3 transcriptome data (UCSC) with topHat2 and allowing only up to 3 mismatches for each alignment. Non-unique alignments were excluded. A custom python script was used to map the beginning of each read to positions in the genome to generate bedgraph files (see included files). Custom MATLAB® script was then used for analysis and is available as a supplement at IST Austria Data Repository, http://dx.doi.org/10.15479/AT:ISTA:45.

### Pause calling

Locations in coding regions with read counts greater than 4 standard deviations above the local (201 bp) mean were identified as pauses. Analysis was also performed with a cutoff of 2 or 6 standard deviations with no significant changes in the qualitative conclusions. See also S3 Table.

### Gene sequences considered

The analysis shown in Figs 1A, 2 and 3B is done considering all genes. For the backtracking analysis shown in Fig 4, all genes were considered; however, only such *dst1Δ* pause sites, for which there exists a corresponding pause site in WT dataset 0–15 nt upstream, were considered. For the rest of the analysis, in order to decrease the influence of false negatives in calculating the predictive power (a pause site cannot be found unless the region is transcribed), only the data for highly expressed genes were used, as determined by dividing the total NET-seq reads for the gene by the gene length; we took the genes with a higher than average density of reads, meaning we perform the analysis on ~25% of the entire dataset. As no selection on genes enriched for nucleosomes was performed, to ensure a fair comparison with nucleosome model, we computed the AUC for the nucleosomes on the reduced and on the total datasets and observed little difference; if all genes are considered, the AUC of the nucleosome model for *dst1Δ* is 0.54, and the AUC of the TEC energy model is 0.63 for the WT dataset and 0.60 for the *dst1Δ* dataset.

For reasons of technical ease, all the calculations of determining the average pause profile and determining the AUC are calculated on a concatenated set of sequences comprised of all the considered genes. Although the points at which these concatenations occur would not occur in the actual yeast genome, these edge effects constitute a very small fraction of the data.

### Predicting RNAP pausing from sequence using a position weight matrix

To determine the DNA sequence specificity near pause sites, we aggregated and aligned the sequences surrounding each pause site and counted the frequency of each nucleotide at each position relative to the pause site. The nucleotide frequencies at each of the positions were computed and allowed construction of a Position Frequency Matrix (nucleotide frequencies at each position around a pause site). This matrix was transformed into a Position Weight Matrix (PWM) with a log transformation of the ratio of each frequency to the overall frequency of nucleotides in the genes considered.

The PWM was then used to score all query sequences from transcribed regions: for each the nucleotide in the query sequence, the identity of the nucleotide was looked up in the PWM table and the scores were summed to calculate a final score for each query sequence. This score assesses how close the query sequence is to the consensus sequence.

To calculate the PWM including information from nearest neighbor sequences, the same procedure was performed with *dinucleotide* (instead of single nucleotide) information at each of the positions instead of single nucleotide. This idea was previously used in Zhao et al. [35].

### Basepairing energy calculation

Previous work has shown that the energy required to melt a complementary nucleic acid duplex can be determined by its sequence. Both the identity of each base and its nearest neighbor, influence the energy required to melt the duplex. By adding up the values from the dinucleotide entries in S1 and S2 Tables (taken from [25] and [26]) the energy required to melt any sequence can be determined. The RNA:DNA and DNA:DNA basepairing energies were assigned to each position in the coding sequence using energy values from nearest neighbor model based on experimental results, previously reported in literature [25,26], assuming the temperature of 303 K. These values include both base *pairing* and base *stacking* interactions.

For a given 2-nt sequence, the basepairing energy was assigned to the position more 3’ of the two, as judged from the coding strand. For RNA:DNA, it matters which nucleotides are in RNA and which in DNA—the coding strand was considered to be in RNA and the template strand in DNA form.

The RNA:DNA basepairing energy dataset [26] was originally determined in conditions of 1 M NaCl, 10 mM Na_2_HPO_4_, 1 mM Na_2_EDTA, pH 7.0. The DNA:DNA basepairing energy dataset [25] was calculated unifying previous reports from multiple different conditions and reported to be in good agreement with DNA:DNA basepairing dataset measured by Sugimoto et al [40], which was measured in the same conditions as the RNA:DNA dataset.

### Transcriptional mismatch analysis

The basepairing energies for RNA:DNA mismatches were calculated for the temperature of 303 K using energy values previously reported in the literature [41] for rAᐧdA, rCᐧdC, rGᐧdG and rUᐧdT basepairs in the conditions of 1 M NaCl, 10 mM Na_2_HPO_4_, 0.5 mM Na_2_EDTA, pH 7.0. These energy values are available only for one out of four possible mismatches for given deoxyribonucleotide, hence our analysis does not cover the entire spectrum of possible transcriptional errors. These energy values reflect the energy contribution of internally mismatched RNA:DNA hybrid (as opposed to terminal mismatch), thus for analysis in S1 Fig we calculated TEC stabilities assuming a transcriptional error at the penultimate 3’ ribonucleobase. This corresponds to a situation when RNAP is able to overcome the misalignment of template and RNA hybrid and to add the following ribonucleotide correctly to the nascent strand.

We have calculated the mismatched TEC stability difference (see below) for each site in the genome that was considered in the pausing analysis of non-mismatched sequences. Using the pausing vs. TEC stability difference dependence inferred from the *dst1Δ* NET-seq data depicted in Fig 3C we have estimated the pausing in case of an RNA mismatch (S1B and S1C Fig) and using the backtracking vs. TEC stability difference dependence inferred from the combined *dst1Δ* and WT dataset depicted in Fig 4B we have estimated the pausing in case of an RNA mismatch (S1D and S1E Fig.)

### TEC energy difference calculation

The TEC stability is defined as RNA:DNA basepairing energy for a specific nucleotide sequence minus the DNA:DNA basepairing energy for that same sequence (Fig 3A).

For the *dst1Δ* strain, the TEC stability difference at position *i* was calculated by taking the TEC stability when bases *i-3* (upstream) through *i+4* (downstream) were in the RNA:DNA hybrid and subtracting it from the TEC stability when bases *i-10* through *i-3* (upstream) are in RNA:DNA hybrid. For the WT strain, the TEC stability difference was calculated by taking the TEC stability when bases *i-14* through *i-7* were in RNA:DNA hybrid and subtracting it from the TEC stability when bases *i-7* through *i* were in RNA:DNA hybrid.

These basepairs were chosen to 1) match the length of the RNA:DNA duplex (8 bp) and 2) be consecutive and 3) change in sign at the site where the basepairing energy profile (colored lines, Fig 2C and 2E) crossed the line of null expectation (gray lines, Fig 2C and 2E). Fitting of these parameters, including the length of the RNA:DNA duplex or considering overlapping or non-consecutive stretches, resulted in the same qualitative results and did not sufficiently improve the prediction power of our model to warrant the introduction of fitting parameters.

### Receiver operating characteristic

To determine the AUC value for various models, the pause prediction true positive rate was plotted against the pause prediction false positive rate, for various threshold values of the binary classifier specified by the model and the area under the resulting curve was summed. The true positive rate is defined as the number of correctly identified positives over the total number of positive cases, and the false positive rate as the number of false positives over the number of all negative cases.

### Nucleosomes

The positions of unique nucleosomes were taken from supplementary table S2 from Brogaard et al. [19].

To create the AUC values for nucleosomes, the probability of pausing as a function of the distance to the nearest nucleosome (up to 500 bp) was computed as in Fig 1A. These pause probabilities were then used as a score for each position in the genome. Positions with nucleosomes greater than 500 bp away were assigned a score equal to the mean pause probability.

For WT pauses that were considered in Fig 4, we checked whether long backtracking could be predicted by these same scores and found an AUC of 0.53.

### Statistical analysis

The statistical analysis (Fig 2B and 2D and S1A Fig) to determine p<0.001 was performed by selecting a number of random sites (in the set of genes considered in each of the respective analysis) equal to the number of pause sites in each of the respective analysis, and averaged for the quantity in question plotted on y-axis the same way this has been done for the pause sites. This procedure was performed 1000 times and the most extreme values for each position on x-axis were plotted as a gray band denoting the p<0.001 limits.

## Supporting information

S1 Fig Decreased TEC stability due to transcriptional error is predicted to result in ~5-fold more pausing followed by backtracking compared to a situation without an error. **A.** Distribution of TEC stabilities after transcriptional error (red) have been calculated considering an RNA:DNA mismatch at penultimate 3’ RNA base using previously reported thermodynamic values [41] (cf. *Materials and Methods*). **B.** The odds of pausing during forward movement of RNAP as a function of TEC stability difference, as inferred from NET-seq (green, cf. Fig 3C inset) was used to infer odds of pausing in case of RNA:DNA mismatch (red). **C.** Predicted odds of pausing for mismatched RNA:DNA hybrid from the left part of panel (B.) transformed into the fraction of sites predicted to have a pause. **D.** The odds of a backtrack following a transcriptional pause being a long backtrack as a function of TEC stability difference, as inferred from NET-seq (green, cf. Fig 4B) was used to infer the odds of long backtracking following a pause in case of RNA:DNA mismatch (red). **E.** Predicted odds of long backtracking following a pause for mismatched RNA:DNA hybrid from the left part of panel (D.) transformed into the fraction of backtracks that are long backtracks (2–15 bases).
(PDF)

S2 Fig TEC energy model also predicts pause locations in bacteria. **A.** The average profile of TEC stability around initial RNAP arrest site as determined from bacterial NET-seq [31]. **B.** For each location in the transcriptome, the TEC stability energy difference is calculated and those locations with the same TEC stability energy difference are grouped. The fraction of locations with a pause site is then plotted against the TEC stability energy difference. **C.** Receiver operating characteristic curves and AUC values for the energy model and the PWM model (with dinucleotide frequencies) of initial RNAP pausing for bacterial NET-seq.
(PDF)

S1 Table Values for energy (kJ/mol) required to break a DNA:DNA basepair, used in RNAP pausing model. Energy values were calculated for the temperature of 303 K based on nearest neighbour model from Table 2 in [25].
(PDF)

S2 Table Values for energy (kJ/mol) required to break an RNA:DNA basepair, used in RNAP pausing model. Energy values were calculated for the temperature of 303 K based on nearest neighbour model from Table 3 in [26].
(PDF)

S3 Table Key Numbers for the main data set. (PDF)
