# Supplementary figures and images for: Sequence-specific thermodynamic properties of nucleic acids influence both transcriptional pausing and backtracking in yeast
Source: PLoS One. 2017 Mar 16;12(3):e0174066. doi: 10.1371/journal.pone.0174066 (PMC5354634; doi:10.1371/journal.pone.0174066)

Figure S1

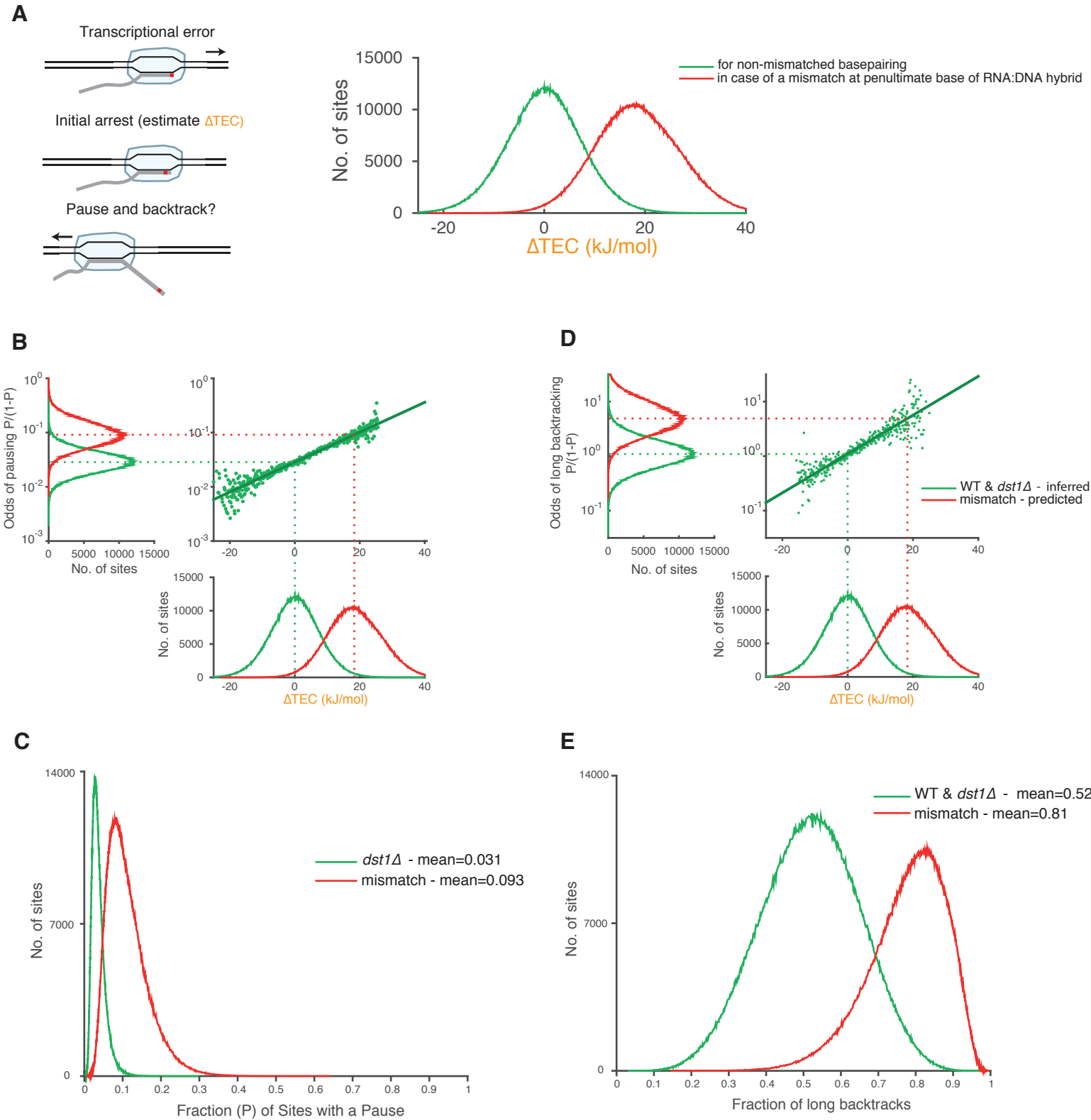

Supplement: S1 Fig — A. Distribution of TEC stabilities after transcriptional error (red) have been calculated considering an RNA:DNA mismatch at penultimate 3’ RNA base using previously reported thermodynamic values [41] (cf. Materials and Methods). B. The odds of pausing during forward movement of RNAP as a function of TEC stability difference, as inferred from NET-seq (green, cf. Fig 3C inset) was used to infer odds of pausing in case of RNA:DNA mismatch (red). C. Predicted odds of pausing for mismatched RNA:DNA hybrid from the left part of panel (B.) transformed into the fraction of sites predicted to have a pause. D. The odds of a backtrack following a transcriptional pause being a long backtrack as a function of TEC stability difference, as inferred from NET-seq (green, cf. Fig 4B) was used to infer the odds of long backtracking following a pause in case of RNA:DNA mismatch (red). E. Predicted odds of long backtracking following a pause for mismatched RNA:DNA hybrid from the left part of panel (D.) transformed into the fraction of backtracks that are long backtracks (2–15 bases). (PDF) [file pone.0174066.s001.pdf]

Figure S2.

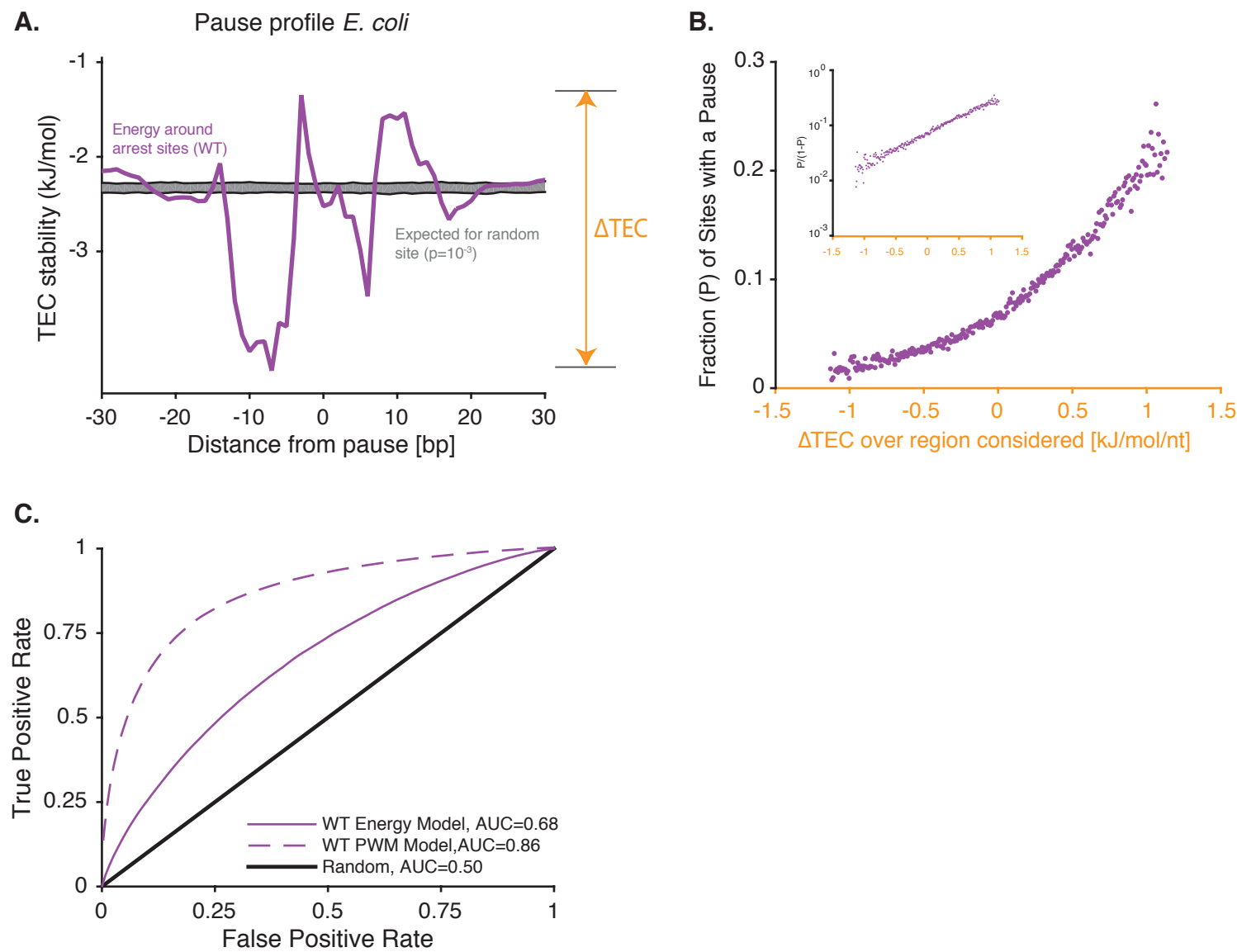

Supplement: S2 Fig — A. The average profile of TEC stability around initial RNAP arrest site as determined from bacterial NET-seq [31]. B. For each location in the transcriptome, the TEC stability energy difference is calculated and those locations with the same TEC stability energy difference are grouped. The fraction of locations with a pause site is then plotted against the TEC stability energy difference. C. Receiver operating characteristic curves and AUC values for the energy model and the PWM model (with dinucleotide frequencies) of initial RNAP pausing for bacterial NET-seq. (PDF) [file pone.0174066.s002.pdf]
