# Supplementary material for: Sequence-specific thermodynamic properties of nucleic acids influence both transcriptional pausing and backtracking in yeast
Source: PLoS One. 2017 Mar 16;12(3):e0174066. doi: 10.1371/journal.pone.0174066 (PMC5354634; doi:10.1371/journal.pone.0174066)
Supplement: S1 Table — Energy values were calculated for the temperature of 303 K based on nearest neighbour model from Table 2 in [25]. (PDF) [file pone.0174066.s003.pdf]

S1 Table. Values for energy (kJ/mol) required to break a DNA:DNA basepair, used in RNAP pausing model. Energy values were calculated for the temperature of 303 K based on nearest neighbour model from Table 2 in [25].

**3' base** / **Nearest 5' neighbour**	**A**	**C**	**G**	**T**
**A**	4.95	7.15	6.41	4.22
**C**	6.37	8.26	9.92	5.52
**G**	5.71	10.06	8.26	6.27
**T**	3.10	6.59	7.26	4.95
