# Supplementary material for: Sequence-specific thermodynamic properties of nucleic acids influence both transcriptional pausing and backtracking in yeast
Source: PLoS One. 2017 Mar 16;12(3):e0174066. doi: 10.1371/journal.pone.0174066 (PMC5354634; doi:10.1371/journal.pone.0174066)
Supplement: S2 Table — Energy values were calculated for the temperature of 303 K based on nearest neighbour model from Table 3 in [26]. (PDF) [file pone.0174066.s004.pdf]

Table S2. Values for energy (kJ/mol) required to break an RNA:DNA basepair, used in RNAP pausing model. Energy values were calculated for the temperature of 303 K based on nearest neighbour model from Table 3 in [26].

3' RNA base<br>Nearest 5' RNA neighbour	A	C	G	U
**A**	4.87	9.09	8.28	4.43
**C**	4.57	9.50	8.49	4.31
**G**	5.90	11.79	13.11	5.25
**U**	3.22	6.95	7.51	1.97
