# Supplementary material for: Sequence-specific thermodynamic properties of nucleic acids influence both transcriptional pausing and backtracking in yeast
Source: PLoS One. 2017 Mar 16;12(3):e0174066. doi: 10.1371/journal.pone.0174066 (PMC5354634; doi:10.1371/journal.pone.0174066)
Supplement: S3 Table — (PDF) [file pone.0174066.s005.pdf]

Table S3. Key Numbers for the main data set

**Total number of pauses**	**Standard deviation above the local mean**		
	**2**	**4**	**6**
**WT**	2578984	301363	64186
dst1Δ	2059994	276521	55043
***dst1Δ* that are also WT (Percent)**	43%	13%	12%
**WT that are also *dst1Δ* (Percent)**	35%	12 %	10%
